# Sudden Infant Death Syndrome – Role of Trigeminocardiac Reflex: A Review

**DOI:** 10.3389/fneur.2016.00221

**Published:** 2016-12-05

**Authors:** Gyaninder Pal Singh, Tumul Chowdhury, Barkha Bindu, Bernhard Schaller

**Affiliations:** ^1^Department of Neuro-Anesthesiology and Critical Care, All India Institute of Medical Sciences, New Delhi, India; ^2^Department of Anesthesiology and Perioperative Medicine, University of Manitoba, Winnipeg, MB, Canada; ^3^Department of Research, University of Southampton, Southampton, UK

**Keywords:** sudden infant death syndrome, trigeminocardiac reflex, diving reflex, oxygen-conserving reflex, bradycardia, asystole, smoking, prenatal nicotine exposure

## Abstract

Sudden infant death syndrome (SIDS) is an unexplained death in infants, which usually occurs during sleep. The cause of SIDS remains unknown and multifactorial. In this regard, the diving reflex (DR), a peripheral subtype of trigeminocardiac reflex (TCR), is also hypothesized as one of the possible mechanisms for this condition. The TCR is a well-established neurogenic reflex that manifests as bradycardia, hypotension, apnea, and gastric hypermotility. The TCR shares many similarities with the DR, which is a significant physiological adaptation to withstand hypoxia during apnea in many animal species including humans in clinical manifestation and mechanism of action. The DR is characterized by breath holding (apnea), bradycardia, and vasoconstriction, leading to increase in blood pressure. Several studies have described congenital anomalies of autonomic nervous system in the pathogenesis of SIDS such as hypoplasia, delayed neuronal maturation, or decreased neuronal density of arcuate nucleus, hypoplasia, and neuronal immaturity of the hypoglossal nucleus. The abnormalities of autonomic nervous system in SIDS may explain the role of TCR in this syndrome involving sympathetic and parasympathetic nervous system. We reviewed the available literature to identify the role of TCR in the etiopathogenesis of SIDS and the pathways and cellular mechanism involved in it. This synthesis will help to update our knowledge and improve our understanding about this mysterious, yet common condition and will open the door for further research in this field.

## Introduction

Sudden infant death syndrome (SIDS) is defined as the sudden unexplained death of a seemingly healthy child less than 1 year of age, usually during sleep. For the diagnosis of SIDS, the death should remain unexplained even after the autopsy, investigation of mortality scene, and review of clinical history (1). SIDS remains a leading cause of death in infants between ages of 1 month and 1 year. The incidence of SIDS varies between regions and among racial and ethnic subgroups (2, 3). It is a multifactorial disorder, the cause of which is still not fully elucidated. The exact cause of death in SIDS remains unclear; however, the exaggeration of parasympathetic activity and cardiorespiratory response to hypoxia has been suggested as a possible underlying mechanism (4–9). In addition, postnatal age, gestational age at birth, and level of arousability are also linked with SIDS (10, 11).

Infants who succumb to SIDS typically experience a severe bradycardia, which is the most shared and predictive event in infants monitored for life-threatening incidents (12, 13). It may be preceded or is accompanied by centrally mediated apnea. Such abnormal and exaggerated response to sensory trigeminal nerve stimulation has also been implicated in the etiopathogenesis of SIDS (14, 15).

Stimulation of trigeminal nerve leads to consecutive reflex bradycardia, hypotension, apnea, and gastric hypermotility, commonly known as the trigeminocardiac reflex (TCR). This reflex is most often transient, but sometimes may be pronounced and sustainable, particularly, in infants. The diving reflex (DR) (a subtype of TCR) is triggered as a result of stimulation of one of the sensory branches of the trigeminal nerve and leads to inhibition of cardiorespiratory center, thereby causes bradycardia and apnea (16–19). An exaggerated response to hypoxia (i.e., augmented TCR response) causing lethal bradycardia and apnea can be accused of sudden death in the victims of SIDS. In this article, we reviewed the available literature on SIDS to identify the evidence and explore the role of TCR in the pathogenesis of SIDS.

## Trigeminocardiac Reflex

Trigeminocardiac reflex has been classified into various subtypes including central, peripheral, and ganglionic TCR (17, 20–24). The central TCR is triggered by the stimulation of the intracranial part of trigeminal nerve proximal to Gasserian ganglion, and the peripheral TCR is triggered by the stimulation of the ophthalmic, maxillary, or mandibular branches of trigeminal nerve (25–30). TCR triggered due to the direct stimulation of Gasserian ganglion is classified as a separate entity (19).

## Pathway of TCR

**Figure 1:**
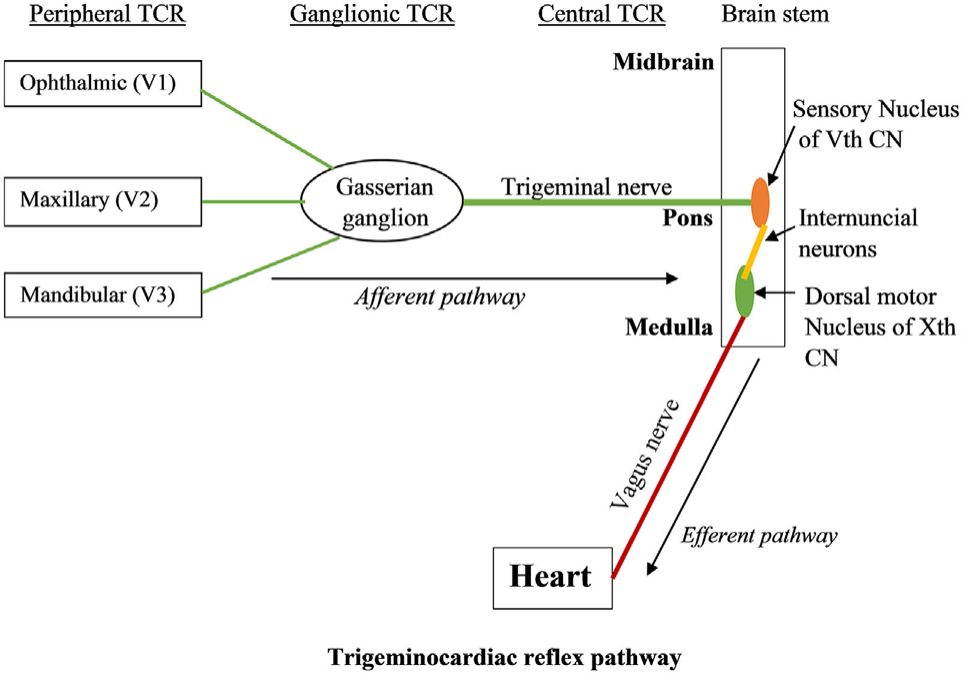
**The trigeminocardiac reflex pathway**.

The branches of the trigeminal nerve, Gasserian ganglion, the sensory nucleus of the trigeminal nerve forms the afferent pathway of the reflex (26, 31–33) (Figure 1). The short internuncial nerve fibers of the reticular formation connect the afferent pathway to the efferent pathway, which is predominantly formed by the parasympathetic neurons of the dorsal motor nucleus of the vagus nerve and nucleus ambiguus (19). Animal studies have shown the involvement of several other brainstem nuclei in the TCR pathway, which includes trigeminal nucleus caudalis, paratrigeminal nucleus, parabrachial nucleus, rostral ventrolateral medulla oblongata, and dorsal medullary reticular field (34–36). Also, various subtypes of TCR show a difference in their reflex arc. While peripherally originated TCR is relayed *via* the spinal nucleus of the trigeminal nerve to the Kölliker–Fuse nucleus, the centrally originated TCR is conveyed *via* the nucleus of the solitary tract to the lateral parabrachial nucleus (35).

Activation of the sympathetic nervous system has been implicated for the other less common manifestations of TCR, such as tachycardia and hypertension, which are seen in some subtypes of TCR. Studies have revealed that stimulation of the anterior ethmoidal nerve in the nasal mucosa (peripheral TCR) may simultaneously activate the sympathetic and vagal responses. This may result in parasympathetically mediated bradycardia along with sympathetically mediated peripheral vasoconstriction and hypertension (37, 38). In contrast to this, the centrally stimulated TCR manifests as bradycardia and hypotension due to activation of cardioinhibitory vagal response, whereas the ganglionic TCR is clinically present as either increase or decrease in heart rate (bradycardia/tachycardia) and blood pressure (hypotension/hypertension) (39). These varied presentations of TCR are due to co-activation of parasympathetic and sympathetic nervous system (39).

## Diving Reflex as a Subtype of TCR

The DR is a powerful autonomic reflex that manifests as the reflex bradycardia, apnea, peripheral vasoconstriction, and hypertension triggered by submersion of a face in cold water through branches of the trigeminal nerve (40). Both TCR and DR are phylogenetically oxygen-conserving reflexes, and researchers reveal a similar reflex arch in both (41). Thus, DR appears to be another subtype of TCR (39). The difference between DR and peripheral TCR shows different effect on blood pressure. Although peripheral TCR causes normotension or hypotension, DR leads to hypertension. This is due to intense peripheral vasoconstriction caused by more strong sympathetic stimulation during DR than during peripheral TCR (42). This reflex also persists in humans and is probably inherited from diving birds and amphibians (18, 32, 43–45). It is particularly prominent in infants and manifests as severe bradycardia upon a single submersion of the face in the water (46, 47). Activation of this reflex due to the stimulation of sensory trigeminal fibers over the face and nasal mucosa causes apnea, a sudden drop in heart rate (due to parasympathetic activation), and a gradual increase in blood pressure as a result of peripheral vasoconstriction (due to increase in sympathetic tone) (44, 45). Also, there is a contraction of spleen releasing erythrocytes in the circulation (48, 49). Thus, the blood flow is redirected to vital organs (the brain and the heart) from the periphery and visceral organs. The heart rate is reduced, thereby lowering O_2_ requirement of myocardium and the blood flow to the brain is increased without an increase in the cerebral metabolic oxygen demand. Thus, DR is a protective oxygen-conserving reflex (18, 32, 44, 45). However, an exaggerated response that results in profound bradycardia can sometimes prove harmful or even fatal (14, 15, 50) and is often said to be associated with sudden death in infants.

## Role of TCR in Sudden Infant Death Syndrome

Sudden infant death syndrome is the leading cause of death in the postneonatal period (12, 13, 51, 52). The exaggeration of parasympathetic activity and cardiorespiratory response to hypoxia has been suggested as the possible mechanism for these events (4–9). Similarly, trigeminal air-stream stimulation (TAS) model also showed that the TAS may induce apnea and bradycardia in premature infants (53). In infants monitored for apparent life-threatening events, severe bradycardia was the most prevalent and predictive event seen in infants who succumbed to SIDS (12, 13, 54), and hypoxia is a frequent event that precedes death in infants of SIDS (55).

Interestingly, the laryngeal chemo-reflex (LCR), a protective mechanism, causes closure of the glottis, coughing, and apnea during aspiration of the fluid into larynx/trachea and, therefore, has also postulated as one of the causes of SIDS (56, 57). On the other hand, DR, a subtype of TCR, has also been implicated to have a role in SIDS (14, 15, 58). The TCR is regulated by many brainstem nuclei and endogenously modulated by many neurotransmitters, the important one being serotonergic (5-HT), cholinergic (ACh), and nicotinergic (42, 59). Abnormalities in the modulation of these neurotransmitters along with defect in brainstem nuclei maturation may lead to exaggerated TCR response. Therefore, we summarize pieces of evidence in four hypotheses.

### Serotonergic Hypothesis

Abnormalities of serotonergic neurons have been observed in victims of SIDS (60–67). These victims had a higher number of 5-HT neurons in the medulla and cerebrospinal fluid (68, 69). Also, these medullary 5-HT neurons have been proposed to act as central respiratory chemoreceptors that are involved in the facilitation of respiration in response to hypoxic episode and generation of respiratory rhythm (70–73). These observations suggest that medullary 5-HT dysfunction may result in loss of respiratory and autonomic response to hypoxia and hypercarbia leading to sudden death in SIDS victims during sleep. Interestingly, the 5-HT1A-binding density was more reduced in males compared to females’ SIDS victims, which also explains why males are more vulnerable to SIDS (68, 74). Notably, these conditions (hypoxia, hypercarbia, and male gender) are also common risk factors for inciting the TCR. In animal experiment models, investigators have shown that the serotonin modulation is linked with TCR mechanism that further explains the possible role of TCR in SIDS (42, 75).

### Cholinergic Hypothesis

The decrease in cholinergic receptors density, as well as binding dysfunction of cholinergic receptors, has also been implicated as a risk factor for SIDS. Investigators have found a reduction in some choline acetyltransferase (ChAT) neurons as well as their binding capacities in hypoglossal nucleus and dorsal motor nucleus of vagus in SIDS cases (76–84). Also, hypoplasia of the arcuate nucleus has also been observed in these infants (76, 85, 86). These findings suggest a specific defect in cholinergic neurons in the brainstem of SIDS infants, which could cause abnormal control of cardiovascular and respiratory functions in these babies and contribute to the etiology of SIDS (76). They observed that cholinergic neurons endogenously inhibit the excitatory glutamatergic transmission to parasympathetic cardiac vagal neurons in response to trigeminal nerve stimulation *via* mAChRs. Neostigmine (an acetylcholinesterase inhibitor) significantly inhibited, whereas atropine (muscarinic receptor antagonist) enhanced this transmission, thus demonstrating the role of muscarinic (m4 type mACh) receptors. A decreased cholinergic activity could result in reduced inhibition of excitatory neurotransmission to cardiac vagal neurons in response to trigeminal nerve stimulation and thus an exaggerated TCR response in SIDS infants (59). Cholinergic receptors also play a significant role in sleep-dependent changes. The cholinergic neurotransmission in the brainstem is an important integral component of rapid eyeball movement sleep generation (87, 88). Change in cholinergic receptor activity is associated with potentiation of TCR and trigeminally evoked respiratory suppression (89) as well as with altered sleep–awake cycles in infants both of which are also seen in victims of SIDS. Studies have identified incomplete and less frequent arousal from sleep in response to hypoxia in SIDS victims. Kato et al. studied the characteristics of arousal from sleep in 16 infants who were being monitored for some days or weeks before they died of SIDS. The polygraphic sleep recordings of these infants were compared with those of matched control infants. The result of this study showed significantly fewer cortical arousal (complete arousal) in an infant who eventually died of SIDS later than in the control infants. Victims of SIDS had more frequent and longer duration of subcortical arousal (incomplete arousal) than controls. This study suggested an incomplete arousal process from sleep in infants who succumb to SIDS (90). Sensory stimulation of the trigeminal nerve during REM sleep has been shown to cause REM sleep-associated respiratory failures in SIDS infants (89, 91).

### Nicotine Hypothesis (Mixed Model)

Prenatal exposure of the fetus to nicotine alters the density and binding capacity of serotonin and cholinergic receptors (61, 63, 68, 80, 81, 92, 93) and is one of the major risk factors contributing to SIDS (94, 95). Gorini et al. used a rat model to study the effects of prenatal nicotine exposure in the offsprings of the mothers who were exposed to clinically significant nicotine levels during gestation (75). The results of this study showed an exaggerated TCR response in animals exposed to nicotine during the prenatal period. They observed that prenatal exposure to nicotine significantly facilitates excitatory glutamatergic neurotransmission to cardiac vagal neurons in the nucleus ambiguus upon stimulation of trigeminal sensory afferents compared to their unexposed counterparts. The prenatal nicotine exposure also enhanced the endogenous serotonergic facilitation of TCR. All these effects thus lead to heightened TCR response (75). Also, a reduction in the number and function of AChRs has also been found in infants exposed to prenatal nicotine. Fetal exposure to nicotine suppresses mRNA expression and thus decreases brainstem mAChR binding. This again contributes to exaggeration of TCR by reduced inhibition of cardiac vagal neurons. Exposure to nicotine during the prenatal period facilitates modulation of inhibitory and excitatory pathways to the vagal nucleus in response to hypoxia or hypercapnia (96, 97). Prenatal exposure to nicotine decreases inhibitory GABAergic signals to the vagal nucleus during hypoxia (98) as well as hypercapnia (99). This decreased inhibitory GABAergic inputs to vagal nucleus cause increase in vagal activity to heart, thereby causing severe and sometimes lethal bradycardia in these animals (100–102). These findings suggest the likely cellular mechanism that causes an exaggerated response and pronounced bradycardia in victims of SIDS. Fetal exposure to nicotine also causes dysfunction of brainstem monoaminergic pathway. It leads to downregulation of 5-HT receptors and enhancing the risk of death due to SIDS (85). Prenatal nicotine exposure modulates 5-HT receptors in areas of brainstem regulating cardiorespiratory function that results in exaggerated TCR response and lethal outcome (75, 103).

### Other Hypotheses

Frequent developmental abnormalities in the brain stem, particularly in the arcuate nucleus, have been identified in SIDS (85, 86, 104–106). The arcuate nucleus is an important cardiorespiratory center in the medulla and hypoplasia of this nucleus has been detected in over 50% of infants dying of SIDS (105). Alterations in another brainstem nucleus have also been demonstrated (85, 107–112). Some of these nuclei (e.g., nucleus ambiguus, parabrachial nucleus) also participate in the reflex arc of TCR (40, 42). Besides, Lavezzi et al. observed an association between tobacco use and decreased in the functional activity of trigeminal nucleus that can trigger sudden death in babies (108). On the other hand, SIDS may occur due to a lack of sufficient development and plasticity of glutamatergic synapses (insufficient glutamate signaling) in the mesencephalic nucleus of the trigeminal nerve and reticular formation of the brainstem (113). All these findings thus suggest the role of developmental defects (i.e., neuronal deficiency and immaturity) in the brainstem nuclei regulating cardiorespiratory and other autonomic function in infants who die of SIDS. Therefore, the TCR may be a missing link in the etiopathogenesis of this subgroup of patients as well.

## Conclusion

Serotonergic or/and cholinergic dysfunction in the brainstem autonomic nuclei causes an exaggerated TCR response and thus culminates in sudden intense bradycardia, apnea, and death and, therefore, can be linked with the etiopathogenesis of SIDS. However, whether the exaggerated TCR response is the cause in all cases of SIDS is a subject for future research.

## Author Contributions

TC made substantial contributions to conception and design, and/or acquisition of data, and/or analysis and interpretation of data, and helped in writing the manuscript. GS participated in drafting and writing the article. BB participated in writing the article. BS participated in writing and gave final approval of the version. All the authors have given final approval for submission of this version.

## Conflict of Interest Statement

The authors declare that the research was conducted in the absence of any commercial or financial relationships that could be construed as a potential conflict of interest.
